# Evolution of the Use of Circulating DNA as a Biomarker in Neoadjuvant Therapy of Breast Cancer

**DOI:** 10.3390/curroncol33080450

**Published:** 2026-07-27

**Authors:** Jannis Tornikidis, Filip Pazdirek, Alan Stolz, Marek Minarik

**Affiliations:** 1 Department of Surgery, Motol University Hospital, 2nd Faculty of Medicine, Charles University, 150 00 Prague, Czech Republic; 2 Department of Analytical Chemistry, Faculty of Science, Charles University, 128 00 Prague, Czech Republic; 3 Elphogene, s.r.o., 161 00 Prague, Czech Republic

**Keywords:** breast cancer, cfDNA, ctDNA, neoadjuvant chemotherapy, prediction, prognosis

## Abstract

**Background:** Breast cancer treatment is often based on multimodal approaches in locally advanced stages typically including neoadjuvant chemotherapy (NACT). There are limited options for the assessment of prognosis and early identification of future non-responders, which has led to the study of circulating cell-free DNA (cfDNA) and its tumor-derived subset, circulating tumor DNA (ctDNA), for potential use as non-invasive markers for prediction of response and prognosis associated with NACT. **Methods:** We have evaluated the literature on approaches to the use of cfDNA and/or ctDNA as potential biomarkers for NACT. Results: Out of 142 references going back to 2010, we found there were 87 original research reports, 39 reviews, 10 clinical trial reports and six case reports. A detailed analysis revealed several distinctive ways that markers were evaluated in a clinical setting. The original studies have focused on cfDNA, especially cfDNA integrity, whereby increasing integrity levels correlate with tumor shrinkage, reductions in proliferation markers, and hence indicate a better prognosis. Similarly, epigenetic alterations have shown promising results, with methylated ctDNA levels decreasing in responders. Further studies demonstrated the utility of ctDNA persistence through the NACT as strongly associated with shorter disease-free and overall survival. The most recent approaches of longitudinal ctDNA monitoring were found to be valuable for early identification of patients at high risk for post-operative recurrence. **Conclusions:** It should be noted that while most reports indicate the important role of circulating DNA in the assessment of prognosis and early detection of recurrence, there is currently only a limited utility in the prediction of eventual neoadjuvant therapy outcomes.

## Simple Summary

Management of locally advanced breast cancer necessitates multimodal treatment, including neoadjuvant chemotherapy (NACT). Current predictive and prognostic markers often fall short in the neoadjuvant context. We have evaluated past and current applications of cfDNA and/or ctDNA in the neoadjuvant setting. The original studies have focused on cfDNA as an indicator of response, particularly cfDNA integrity, whereby higher integrity levels correlate with better prognosis. Similarly, levels of epigenetic methylation of ctDNA were significantly decreased in treatment responders. The use of ctDNA positivity and its persistence were associated with shorter disease-free and overall survival. Finally, approaches based on longitudinal evaluation of ctDNA have demonstrated clinical utility for early identification of patients at high risk for post-operative recurrence. While most reports include assessment of prognosis and detection of recurrence, there is only limited utility in the prediction of outcomes of NACT.

## 1. Introduction

Breast cancer, with an estimated 2.3 million cases diagnosed annually, is the most prevalent malignancy among women (accounting for 23% of all cancers in women) and the second most common cancer overall. Despite its relatively favorable prognosis, it claims the lives of more than 665,000 patients each year [1]. The disease requires a multimodal treatment approach for optimal patient outcomes. Available therapeutic strategies include surgery, chemotherapy, radiotherapy, endocrine therapy, immunotherapy, and targeted molecular therapies. Despite the continuing progress in these treatment modalities, breast cancer survival rates remain disparate across regions. For example, the 5-year survival rates in Asia range between 65% and 89%, attributed to late diagnoses and limited access to advanced therapies [2,3].

Similar to other solid cancers, surgical intervention offers the best prospects for long-term survival. Furthermore, neoadjuvant chemotherapy (NACT), administered preoperatively to improve surgical outcomes, has significantly impacted patient outcomes, particularly in early-stage and locally advanced breast cancer [4,5]. NACT, originally developed to reduce tumor size preoperatively and target micrometastatic disease, has emerged as a cornerstone in the multidisciplinary management of breast cancer, improving survival rates and enabling more conservative surgical approaches. Its systemic effects target undetectable micrometastatic disease, significantly reducing the risk of both local and distant recurrence. Pathological complete response (pCR), defined as the absence of invasive cancer in the breast and lymph nodes post-treatment, is a critical prognostic marker in patients receiving NACT [6]. Studies have demonstrated excellent long-term outcomes in patients achieving pCR, with 10-year relapse-free survival rates reaching 95% in HER2-positive and 83% in estrogen receptor-positive, HER2-negative subtypes. However, despite dual HER2 blockade, a 10% recurrence rate persists in node-positive patients, highlighting the need for enhanced diagnostic precision and therapeutic strategies.

While NACT is generally the preferred treatment option, not all patients respond favorably. Consequently, many are exposed to unnecessary toxicity and treatment delays without clinical benefit. Identifying non-responders by reliable predictors of NACT response is essential to optimize patient selection, personalize treatment regimens, and improve overall prognosis. It is clear that predictive markers need to reflect specific features of the tumor, hence its molecular basis. There are several molecular assays currently employing tumor-specific features and genomic profiles in the management of breast cancer, including HER2DX, MammaPrint, and Oncotype DX. However, while these tests are able to predict recurrence risks and respond to chemotherapy efficiently, they fall short in assessing residual disease in the neoadjuvant context [7,8]. Development and validation of a novel class of molecular biomarkers is therefore highly desirable.

### 1.1. Circulating DNA as a Non-Invasive Biomarker in Solid Cancers

As noted above, identifying reliable predictors of NACT response is essential to optimize patient selection, personalize treatment regimens, and improve overall prognosis. One emerging area of interest is the analysis of DNA fragments circulating in peripheral blood. The DNA in the circulation of patients with solid cancers includes fragments released by cells via physiological processes of apoptosis, necrosis, or active secretion [9]. The amount of cell-free DNA (cfDNA) in plasma reflects these mechanisms and is directly related to the extent of ongoing processes associated with malignant cell proliferation. Moreover, the cfDNA contains a specific subset of fragments derived from malignant cells originating in the tumor. The release of the so-called circulating tumor DNA (ctDNA) into the bloodstream is influenced by tumor biology and treatment effects [10].

ctDNA can be differentiated by tumor-specific genetic mutations or epigenetic alterations with high specificity, and its amount is measured as a minor allele fraction (MAF) relative to the overall cfDNA present in circulation. It has been repeatedly confirmed and reported that the MAF is directly related to the cancer stage as well as the overall tumor burden, reflecting the sum of all malignant lesions currently present in the patient [11]. Treatment-induced cell death, particularly during chemotherapy, results in a transient spike in ctDNA levels, followed by clearance as the tumor burden decreases. The dynamics of ctDNA release can provide insights into the effectiveness of therapy and potential resistance mechanisms [12].

### 1.2. The Influence of Histological (Molecular) Subtype of Breast Cancer on ctDNA Release

The amount of circulating tumor DNA (ctDNA) released into the bloodstream is not the same in breast cancer across individual molecular subtypes. In recent years, it has been shown that the biological properties of the tumor significantly influence the so-called ctDNA shedding, i.e., the intensity of tumor DNA release into the circulation. The highest rate of ctDNA release has been repeatedly described in triple-negative breast cancer (TNBC) [13]. These tumors are characterized by high proliferative activity, significantly higher cell turnover, more extensive necrosis and more often also a higher representation of immune cells in the tumor microenvironment. HER2-positive tumors are also relatively strong “shedders”. Although there are fewer studies available than for TNBC, review papers report that HER2-positive cancers generally show higher ctDNA detectability than hormone-positive (luminal) tumors mag. Higher proliferation, greater genomic instability and higher tumor cellularity probably contribute to a more intense release of tumor DNA into the bloodstream [14]. In contrast, luminal tumors (HR+/HER2−, especially Luminal A) are typical low shedders. Their biological behavior is usually less aggressive, proliferation is lower, and apoptosis and necrosis are less pronounced. ctDNA concentrations tend to be low, especially in early stages of the disease, which is one of the main limitations of liquid biopsy in this group of patients. Detection of minimal residual disease therefore often requires highly sensitive, tumor-informed methods based on a personalized mutation panel. Review articles repeatedly emphasize that luminal carcinomas show the lowest rate of spontaneous ctDNA shedding.

It is important to emphasize that the molecular subtype itself is not the only determinant of shedding. Total tumor mass, the presence of visceral metastases (especially liver metastases), a high Ki-67 proliferation index, tumor vascularization, and the extent of cell death also play a significant role. Therefore, some advanced luminal tumors may produce more ctDNA than small early TNBC. This phenomenon naturally has a direct impact on the required sensitivity of the methods used for scrutinizing the ctDNA.

The aim of this review is to present the current state of the art in the field of research, practical application and validation of the utility of detection of circulating DNA in the management of neoadjuvant therapy of breast cancer.

## 2. Materials and Methods

**Figure 1:**
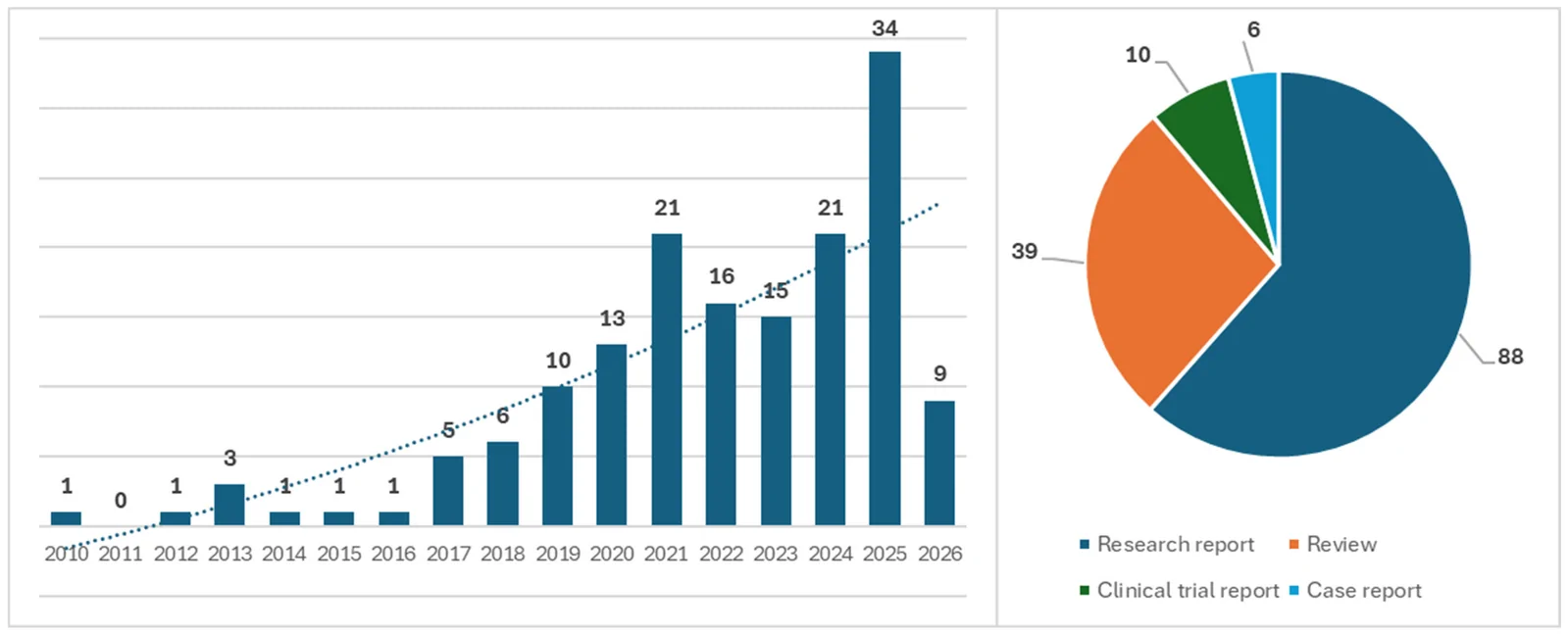
Annual frequency and the publication type of references found on the use of circulating cell-free DNA (cfDNA) or circulating tumor DNA (ctDNA) in neoadjuvant therapy of breast cancer (as of March 2026).

We have performed an extensive literature search using RefMan software utility (Reference Manager version 12, Thomson Reuters, Philadelphia, PA, USA) to scrutinize PubMed Medline (U.S. National Library of Medicine) for publication references related to the topic of the use of circulating DNA as predictive or prognostic markers of neoadjuvant therapy of breast cancer. There was a general search with the subsequent manual categorization of the identified references. The search procedure was directed at the following syntax within the Title/Abstract fields:

„Breast” AND („cancer” OR „carcinoma”) AND („ctDNA” OR „circulating DNA” OR „plasma DNA” OR „cell-free DNA” OR „cfDNA”) AND („neo-adjuvant” OR „neoadjuvant”). The search was performed in early March 2026 and returned a total of 142 references from 2010 up to 2026. The paper types included 87 original research reports, 39 reviews, 10 clinical trial reports and 6 case reports. The publication frequency for the above search query is shown in Figure 1.

The identified original research papers were classified according to distinct areas of focus, technologies or types of markers used.

## 3. Results

**Figure 2:**
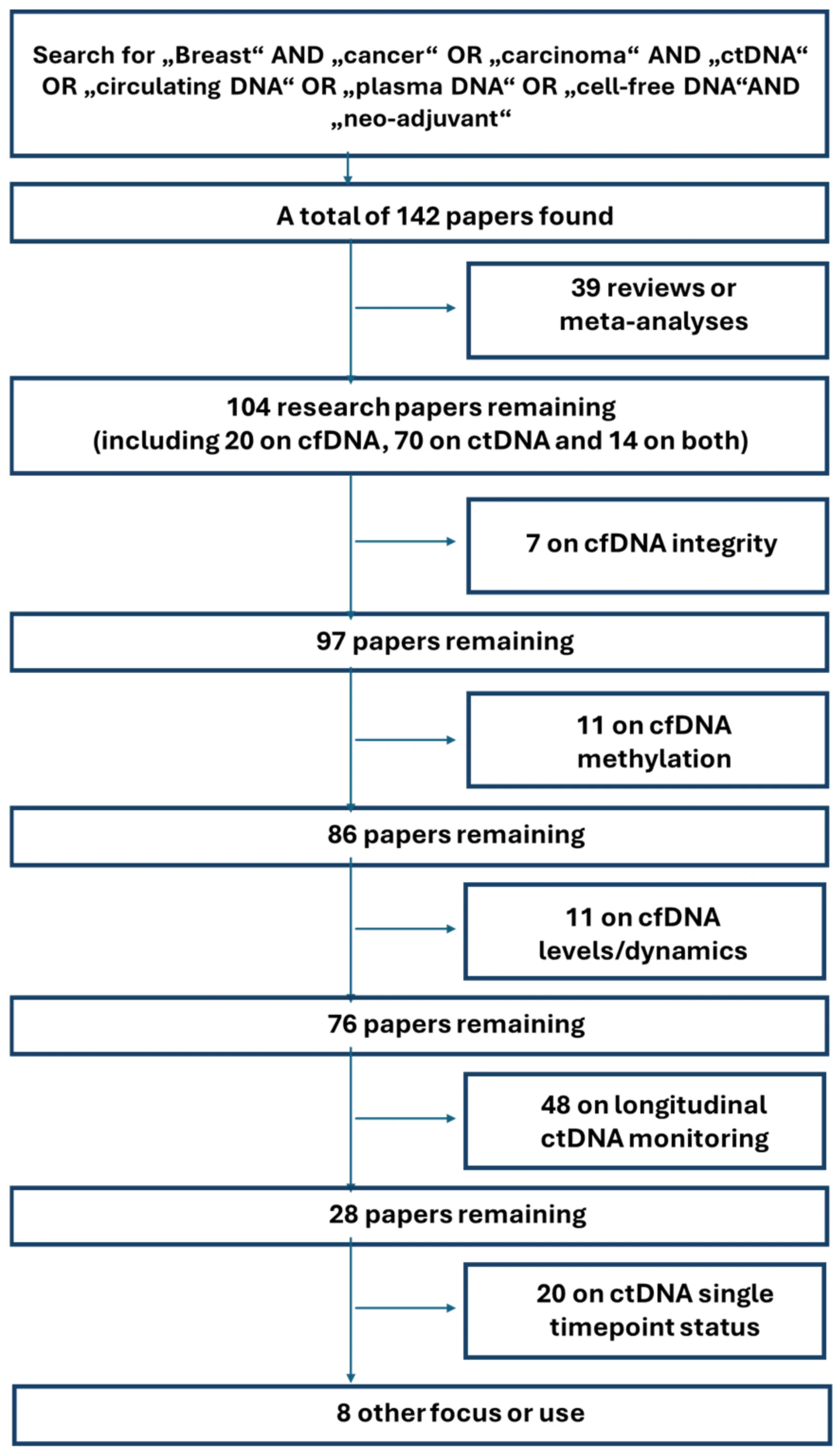
Classification of main topics of found papers on the use of circulating cell-free DNA (cfDNA) or circulating tumor DNA (ctDNA) in neoadjuvant therapy of breast cancer.

Several approaches are currently being explored for clinical utility, as listed in Figure 2. The wide spectrum of topics studied by the identified research papers includes reports on the potential diagnostic utility of cell-free DNA, looking at (i) cfDNA integrity based on specific fragmentation patterns, (ii) cfDNA methylation profiles, or (iii) cfDNA concentration level and its changes throughout the course of the disease and treatment. Additionally, another set of papers is focused on the tumor-derived portion of the circulating DNA. There, the ctDNA is either (iv) used as a single-time-point parameter at baseline or at some point during treatment, or (v) the evolution of ctDNA over time is monitored longitudinally.

## 4. Discussion

### 4.1. cfDNA Concentration Levels

Research on the utility of cell-free DNA (cfDNA) as a biomarker for neoadjuvant chemotherapy (NACT) in locally advanced breast cancer (LABC) has focused on concentration dynamics and its potential to predict treatment response. In 2013, an investigation into a prospective cohort of 50 patients found that baseline levels of total cfDNA did not differ significantly between primary breast cancer patients and healthy controls [15]. This study observed that total cfDNA concentrations actually increased during the course of NACT, but this elevation showed no relationship to the attainment of a pathological complete response (pCR). In 2014, the NEOCENT trial similarly reported that plasma total cfDNA levels rose significantly from baseline to week eight of treatment in patients receiving chemotherapy [16]. This increase was maintained until the time of surgery for the chemotherapy group, whereas levels in the endocrine therapy group returned to baseline. The year 2018 saw further exploration of DNA sources and genomic profiling. A study involving 200 patients confirmed a strong baseline correlation between plasma and urinary DNA concentrations [17]. While mutant DNA copies specifically dropped after surgery, total urinary DNA concentrations increased in a subset of patients during the nine-month monitoring period. Concurrently, genomic characterization of triple-negative breast cancer (TNBC) via cfDNA revealed that a tumor fraction (TFx) of 10% or higher was independently associated with significantly worse metastatic survival, highlighting the prognostic potential of quantifying tumor-derived DNA within total cfDNA [18]. Another prospective study noted a significant decline in total cfDNA concentrations following surgery and chemotherapy, attributing the reduction to a decrease in tumor burden [19]. In 2021, studies continued to highlight the impact of chemotherapy cycles on concentration levels. Analysis of HER2-positive patients showed a slight decrease in the plasma HER2 copy number ratio after NACT, although its clinical utility was deemed somewhat inferior to serum HER2 protein levels [20]. In 2024, comprehensive research identified subtype-specific prognostic values. In the I-SPY2 trial, high pretreatment cfDNA shedding was found to be a significant negative prognostic factor for distant recurrence-free survival in hormone receptor-positive/HER2-negative patients. In the TNBC subtype, a modest negative correlation was observed between cfDNA levels three weeks into treatment and the residual cancer burden at surgery, implying that higher early levels might indicate a better response [16].

Literature focusing on cfDNA concentration levels is summarized in Table 1.

### 4.2. cfDNA Integrity

The absence of utility of baseline cfDNA levels has prompted investigation of additional possibilities for a non-invasive tool in breast cancer NACT settings. Among them, research into cfDNA integrity has evolved significantly over the past decade. It basically refers to assessing the fragmentation pattern of cfDNA and is defined and evaluated as the ratio of the concentration of longer DNA fragments to shorter fragments originating from the same genomic locus [21]. The most frequent approach is to calculate the ratio of repetitive genomic elements ALU 247 (representing longer fragments from non-apoptotic cells) to ALU 115 (representing total circulating DNA) Early findings from 2013 suggested that DNA integrity indices could indicate a complete response (CR) to NACT as early as cycle two and again near the end of treatment, roughly 60–70 days after initiation [22]. This initial report posited that higher integrity levels in the CR group might reflect higher levels of tumor destruction during the therapy process. A subsequent study by the same research group found that while pre-therapeutic integrity levels did not predict the final outcome, the kinetics of the total cfDNA amount (ALU 115) were highly significant, with levels decreasing in patients who achieved CR but increasing in patients whose disease showed no change [23]. Investigations published in 2019 expanded this understanding by demonstrating that mean cfDNA integrity values increased significantly as patients received NACT [24]. This rise in integrity was found to correlate positively with physical tumor shrinkage and a reduction in the proliferation marker Ki67. Furthermore, the research highlighted that patients achieving a pathologic complete response (pCR) exhibited a gradual and steady rise in integrity throughout their treatment, whereas patients who later developed distant metastases showed no significant change in integrity levels. In 2021, a study confirmed that while total cfDNA concentrations were elevated in cancer patients at baseline, these levels decreased significantly after the third cycle of chemotherapy, while the integrity of the remaining circulating DNA increased [24]. This research suggested that serum cfDNA provided a relatively inexpensive and minimally invasive method for evaluating chemotherapy response. By 2022, a report utilized automated electrophoresis to calculate a “cfDI index” after NACT completion, finding that it significantly correlated with the achievement of pCR at surgery [25]. When this integrity index was combined with MRI, the predictive value for a complete response reached 87.5%, while the predictive value for the absence of a response reached 94.7%.

Most recently, research in 2024 has identified cfDNA integrity as a potential very early biomarker, with levels measured just 15 days after starting NACT being significantly higher in patients who eventually achieved pCR [26]. These early measurements also correlated significantly with disease-free survival. That said, however, it should be noted that another investigation from the same year, while confirming a significant decrease in total cfDNA post-treatment, reported no significant change in integrity in their specific cohort [27]. These variations may be attributed to the differences in sample processing, such as isolation procedures, yet the weight of the evidence continues to support cfDNA integrity as a promising tool for real-time monitoring of neoadjuvant therapy response.

Literature aimed at the monitoring of cfDNA integrity is summarized in Table 2.

### 4.3. cfDNA Methylation

Original research into methylated circulating DNA established its role as a dynamic biomarker for monitoring treatment response, with panels including *BRCA1* and *MGMT* demonstrating that total tumor-specific gene methylation levels correlate strongly with tumor volume reduction in responding patients [28]. By 2016, studies of *RASSF1A* promoter methylation confirmed that methylated circulating tumor DNA (met-ctDNA) was a more sensitive marker than conventional protein markers and that its levels significantly decrease after NACT specifically in responders [29]. However, investigators noted a potential “paradoxical increase” in met-ctDNA levels post-NACT—likely due to the slow release or delayed clearance of DNA during tumor cell death. Although this may limit accuracy in predicting a pathological complete response (pCR), met-ctDNA remains significantly associated with overall residual tumor burden.

In 2019, the use of machine learning algorithms further refined these applications, with classifiers based on gene panels like *WNT5A*, *SOX17*, and *KLK10* providing non-invasive prognostic signatures for survival and treatment response [30]. Simultaneously, the integration of multiple breast-specific markers was shown to enhance detection sensitivity, with researchers demonstrating that patients achieving pCR reached methylated cfDNA levels comparable to healthy controls by the fifth month of treatment, while those with residual disease maintained elevated levels [31]. By 2023, universal digital PCR assays targeting both breast-specific methylations (*LMX1B*, *ZNF296*) and cancer-specific markers (*HOXA9*) were developed to evaluate treatment response [32]. While some trials found no significant association between midterm ctDNA status and final pathological response, the persistence of ctDNA identified by methylation markers during treatment continued to be recognized as a significant predictor of poor outcomes.

Recent advancements in 2024 have utilized agnostic, genome-wide approaches like cfMeDIP-seq to reveal distinct patterns in the plasma methylome that clearly differentiate patients who achieve pCR from those with residual disease [33]. In triple-negative breast cancer, post-NACT methylation signal levels were found to be significantly lower in pCR patients than in those with residual disease, with post-treatment methylation status acting as a predictor for both the extent of residual burden and the risk of recurrence [34]. Prospective multicenter studies are now focused on establishing minimal residual disease (MRD) detection through these epigenetic markers to guide treatment escalation or de-escalation [35].

As of 2025 and 2026, methylation-based MRD assays have demonstrated a baseline detection rate exceeding 70% and have been shown to outperform mutation tracking by providing a molecular lead time of several months before clinical relapse [36]. Novel machine learning biosignatures utilizing newly identified markers including *CLDN15*, *MRGPRD* and *ZNF430* have successfully differentiated clinical disease status and predicted treatment response in metastatic settings, underscoring the potential for these liquid biopsy tools to provide real-time, personalized management of breast cancer [37].

Literature on cfDNA methylation is summarized in Table 3.

### 4.4. Assessment of ctDNA Status

Identification of ctDNA from cfDNA mostly relies on the detection of tumor-specific somatic mutations. One of the approaches, referred to as tumor-naïve, relies on sequencing of the cfDNA without any prior knowledge of the presence of mutations within the tumor. This approach is occasionally referred to as non-personalized [38]. With the other, tumor-informed approach, relatively simple mutation detection techniques are often applied, such as allele-specific ARMS (Amplification Refractory Mutation System) PCR or digital PCR, to detect specific somatic mutations that were previously found in the tumor [39]. While the NGS sequencing method is now universally applied for direct processing of patient plasma without the need for tissue samples, the ARMS-PCR and ddPCR are significantly lower in cost, allowing for processing of multiple samples in a longitudinal serial fashion. The principal parameter in the selection of a proper ctDNA detection method is mutation detection sensitivity expressed in MAF mentioned in the Introduction. Following is comparison of various ctDNA detection methodologies in terms of typical sensitivity (%MAF), error rate, and sample turnaround time (TAT):
**Method****%MAF****Err. Rate****TAT****Cost-per-Sample**Sanger sequencing10–20%10^−1^1 daylowQuantitative PCR (qPCR)5–10%10^−3^–10^−2^3–5 hvery lowNGS (amplicon based)1%10^−3^–10^−2^2–3 daysmediumNGS (hybrid capture based)0.1%10^−3^–10^−2^3–5 dayshighAllele specific PCR (ARMS)0.1%10^−3^3–5 hlowNGS (with UMI)0.01%10^−5^4–7 daysvery highDroplet-digital PCR0.01%10^−4^6–8 hlow

The NGS sequencing with UMI was the first method of choice in 2020, when a large secondary analysis of the BRE12-158 trial focused on early-stage triple-negative breast cancer (TNBC) patients with residual disease after NACT [40]. The presence of ctDNA after neoadjuvant therapy and surgery was independently associated with significantly inferior distant disease-free survival (DDFS), disease-free survival (DFS), and overall survival (OS), providing a novel stratification factor for future trials. A 2021 case report using the tumor-informed approach further supported the role of post-surgical liquid biopsy, showing that the detection of a *PIK3CA* mutation in ctDNA following neoadjuvant treatment in TNBC was associated with early relapse and rapid disease progression [41]. The first report directed at ctDNA for the prediction of NACT efficacy in locally advanced breast cancer was published in 2022. The researchers set out to identify the role of specific point mutation biomarkers in baseline ctDNA [42]. As a result, *XRCC1* mutations were significantly associated with high Miller–Payne grades, indicating good treatment efficacy, while *mTOR* mutations were potentially associated with NACT resistance. Prognostic research in 2023 highlighted that end-of-treatment (EOT) ctDNA status in TNBC patients with residual disease provides complementary information to traditional residual cancer burden (RCB) classes [42]. Detection of ctDNA at the completion of all definitive curative therapy was significantly associated with inferior 3-year event-free survival (EFS) and OS, particularly predicting worse outcomes within the RCB-II subgroup. Also in 2023, a post hoc analysis of the phase III PEARLY trial established that baseline ctDNA copy number aberration (CNA) burden, quantified by the I-score algorithm, predicts DFS in stage II-III TNBC patients receiving NACT [43]. A high baseline ctDNA CNA burden was found to be a robust predictor of poor DFS independent of whether the patient achieved a pathologic complete response (pCR), suggesting it could guide the escalation or de-escalation of neoadjuvant strategies. A study in 2024 evaluating early TNBC patients within seven months of completing primary treatment (including neoadjuvant therapy) revealed that 7.7% of patients had detectable residual disease [44]. Analysis indicated a trend where patients with incomplete pathologic response and positive ctDNA were at a much higher risk for reduced progression-free survival. Finally, a relatively recent 2024 analysis integrated ctDNA with traditional biomarkers to assess NACT efficacy, observing that ctDNA levels decreased significantly during therapy in patients who achieved pCR compared to those who did not [45]. It was concluded that monitoring of ctDNA dynamics could serve as a real-time, non-invasive method for assessing treatment response and identifying non-responders early in the therapeutic process.

Literature aimed at the evaluation of ctDNA status is summarized in Table 4.

### 4.5. Longitudinal ctDNA Monitoring

As discussed, a baseline ctDNA status, prior to therapy start, is mainly relevant for assessment of the disease-free as well as overall survival prognosis. For both predicting therapy outcomes as well as the survival, longitudinal monitoring by evaluation of ctDNA dynamics before and during the NACT has emerged as a more suitable tool. In 2015, early investigations established that tracking somatic mutations in plasma after completion of apparently curative treatment can accurately identify minimal residual disease and predict metastatic relapse [46]. By 2017, research in nonmetastatic triple-negative breast cancer (TNBC) demonstrated that ctDNA positivity after just one cycle of neoadjuvant chemotherapy (NACT) is significantly correlated with shorter disease-free and overall survival [47]. Concurrently, studies have proposed that ctDNA levels could serve as the earliest predictors of NACT response [48]. Subsequent investigations in 2019 provided a deeper characterization of ctDNA dynamics, showing that while a dramatic reduction in abundance occurs during treatment, increases in mid-treatment ctDNA levels can serve as early predictors of disease progression and future recurrence [49]. In HER2-amplified patients, ctDNA detection before neoadjuvant therapy was associated with decreased odds of achieving a pathologic complete response (pCR) [50]. Prospective multicenter studies validated that ctDNA detection during follow-up is highly prognostic for relapse across all major subtypes, offering a median lead time of 10.7 months over clinical recurrence [51]. Furthermore, quantitative precision assays demonstrated that ctDNA concentrations after the completion of neoadjuvant therapy are significantly higher in patients with residual disease at surgery compared to those who achieve pCR [52].

Research published in early 2020 demonstrated the utility of tagged targeted deep sequencing (tTDS) to monitor minimal residual disease (MRD) in breast cancer patients receiving NACT [53]. By tracking target mutations in serial plasma samples collected before, during, and after surgery, this method identified deleterious mutations in relapsed cases with an average lead time of six months before clinical recurrence. Another research study directed at TNBC confirmed that ctDNA detection early in treatment and late before surgery is strongly predictive of residual tumor, and its presence at the end of NACT indicates significantly worse relapse-free survival [54]. Identifying specific somatic mutations, such as *KMT2C*, in postoperative samples was highlighted as a potential indicator of recurrence [55]. Longitudinal tracking during NACT also demonstrated that ctDNA outperformed traditional imaging in predicting overall response and provided strong prognostic value for disease-free and overall survival, particularly in estrogen receptor-negative patients [56]. By 2021, comprehensive analyses across all subtypes indicated that patterns of ctDNA change during NACT are significantly correlated with the risk of metastatic recurrence [57]. Detection after NACT remained associated with an increased risk of relapse, predating clinical diagnosis by up to 13 months [58]. In the locally advanced setting, post-operative ctDNA status was found to be the strongest independent prognostic factor for distant metastasis [59]. In 2022, further studies have emphasized that the persistence of ctDNA midway through neoadjuvant systemic therapy negatively predicts response and identifies patients who will not achieve pCR or will have a high residual cancer burden (RCB) [60], while ctDNA detection before surgery and during follow-up continued to be strongly associated with shorter event-free survival [61]. Clinical trial results in 2023 showed that prospective ctDNA surveillance could identify molecular residual disease not visible on imaging, although high rates of metastatic disease were often found upon first detection [62]. Expanded cohort analyses revealed that early ctDNA clearance predicts favorable response in TNBC, while ctDNA positivity at any time point was associated with inferior distant recurrence-free survival in both HR-positive and TNBC subtypes [63]. Finally, ultrasensitive mutation enrichment assays further confirmed a strong association between end-of-therapy ctDNA levels and RCB status [64], while integrated risk models demonstrated that incorporating pretreatment ctDNA levels significantly improves the prediction of pCR [65].

Reports from 2024 and 2025 utilizing tumor-informed assays achieved high sensitivity for ctDNA detection, with detection during monitoring associated with a 100% positive predictive value for future relapse [66,67]. Similarly, epigenomic (methylation) assays also demonstrated high sensitivity for predicting distant recurrence during both neoadjuvant therapy and postoperative surveillance [68]. Undetectable ctDNA during and after NACT was predictive of treatment efficacy and correlated with improved overall survival [69]. Finally, the most recent studies reported in 2026 that focused on HER2-positive patients showed that ctDNA detection after neoadjuvant therapy independently predicted recurrence and could serve as a marker for determining the need for adjuvant T-DM1 therapy [70]. Among baseline ctDNA-positive patients, non-clearance emerged as the strongest independent prognostic factor for disease progression, particularly for TNBC [71]. Lastly, monitoring during neoadjuvant endocrine therapy showed that baseline ctDNA detection is associated with higher pathological stages and RCB scores, while persistent detection prior to surgery predicts a higher risk of distant recurrence [72].

Literature describing longitudinal ctDNA monitoring is summarized in Table 5.

## 5. Conclusions

The integration of circulating DNA as a non-invasive biomarker offers a transformative approach to personalizing neoadjuvant therapy for breast cancer. Research indicates that while baseline cfDNA positivity serves as a significant negative prognostic factor, total cfDNA levels often rise during chemotherapy regardless of response. In contrast, cfDNA integrity indices provide a more refined real-time tool, with rising integrity levels correlating with physical tumor shrinkage and the achievement of a pathological complete response (pCR) following NACT. ctDNA methylation profiles further enhance diagnostic sensitivity, as decreasing methylation levels characterize treatment responders and offer a molecular lead time of several months over clinical relapse. Detection of ctDNA by tumor-specific somatic mutations identifies patients at high risk for recurrence post-therapy. Most significantly, longitudinal ctDNA monitoring demonstrates that early ctDNA clearance is a strong predictor of favorable outcomes, whereas persistent ctDNA positivity during or after treatment is a robust independent indicator of inferior disease-free and overall survival.

## Figures and Tables

**Table 1:** Overview of literature focusing on cfDNA concentration levels.

Paper	Journal	Patient Cohort	Major Finding(s)	Ref.
Bechmann (2013)	*J. Cancer Res. Clin. Oncol.*	50 LABC patients, 50 healthy females, 15 metastatic BC patients	cfDNA not elevated in primary cancer patients. cfDNA levels increased during neoadjuvant chemotherapy, but no relationship to treatment effect or pathological complete response.	[15]
Palmieri (2014)	*Breast Cancer Res. Treat.*	44 ER-rich postmenopausal primary BC patients (22 chemotherapy, 22 endocrine therapy)	cfDNA levels rose significantly from baseline to week 8 in both chemotherapy and endocrine therapy groups. This increase was maintained until surgery for patients in the chemotherapy group, while levels in the endocrine therapy group returned to baseline.	[16]
Liu (2018)	*Clin. Transl. Oncol.*	200 early-stage BC patients receiving NACT, 50 healthy females	Urinary and plasma DNA concentrations at baseline significantly higher in cancer patients than in healthy controls. Total urinary DNA and mutant copies dropped significantly following surgery after neoadjuvant therapy.	[17]
Stover (2018)	*J. Clin. Oncol.*	164 patients with biopsy-proven metastatic TNBC	A cfDNA tumor fraction of 10% or higher was independently associated with significantly worse metastatic survival. Genomic characterization exclusively via cfDNA revealed that metastatic TNBC copy number profiles remarkably mirror those of primary tumors.	[18]
Peled (2020)	*Sci. Rep.*	259 patients suspected with BC (140 positive biopsy, 119 negative/benign results)	Baseline cfDNA concentration could not discriminate between malignant and benign breast disease. However, the reduction of tumor burden through surgery and neoadjuvant chemotherapy was significantly associated with a reduction in total cfDNA levels.	[19]
Qui (2021)	*Breast Cancer*	120 primary BC, 30 metastatic BC, 34 HER2-positive BC patients receiving NAC	Plasma HER2 copy number ratio showed only a small, statistically insignificant decrease after NAC. The clinical utility of the HER2 copy number assay in cfDNA was deemed somewhat inferior to serum HER2 protein levels for treatment monitoring.	[20]
Magbanua (2024)	*Clin. Cancer Res.*	145 HR-positive/HER2-negative, 138 (TNBC)	cfDNA presence is negative prognostic factor for distant recurrence-free survival in HR-positive/HER2-negative patients. For TNBC higher early levels might indicate a better response	[14]

**Table 2:** Overview of literature related to cfDNA integrity.

Paper	Journal	Patient Cohort	Major Finding(s)	Ref.
Lehner (2013)	*Int. J. Clin. Pharmacol. Ther.*	49 LABC	DNA integrity indices (Int 1 and 2) indicate a complete response (CR) as early as Cycle 2 and at the end of treatment.	[22]
Lehner (2013)	*Clin. Chim. Acta*	65 patients with locally confined breast cancer	Kinetics of total cfDNA (ALU 115) monitor response: levels decrease in complete responders but increase in non-responders.	[23]
Wang (2019)	*Transl. Cancer Res.*	29 LABC	cfDNA integrity (cfDI) increases significantly during NACT, correlating with tumor shrinkage, reduced Ki67, and pCR.	[24]
Adusei (2021)	*Med. Sci.*	32 LABC and 32 healthy females	Total cfDNA concentrations decrease significantly while DNA integrity increases after the third cycle of chemotherapy.	[21]
Cirmena (2022)	*JCO Precis. Oncol.*	38LABC and 6 healthy controls	The cfDI index at NACT completion significantly correlates with pCR and enhances the predictive accuracy of MRI.	[25]
Giro (2024)	*Breast Cancer Res. Treat.*	28 LABC	cfDNA integrity (cfDNAI) measured just 15 days after starting NACT is an early biomarker for pCR and disease-free survival.	[26]
Çelik (2024)	*Turk. J. Med. Sci.*	36 LABCand 21 healthy females	cfDNA levels decrease significantly post-treatment, though this specific cohort showed no significant change in cfDNA integrity.	[27]

**Table 3:** Overview of literature on cfDNA methylation.

Paper	Journal	Patient Cohort	Major Finding(s)	Ref.
Sharma (2012)	*Tumor Biol.*	30 LABC	Total gene methylation correlates strongly with tumor volume reduction in responding patients	[28]
Takahashi (2016)	*Clin. Breast Cancer*	87 primary breast cancer patients (stage II–III)	Methylated *RASSF1A* circulating tumor DNA (ctDNA) is a more sensitive marker than CEA or CA 15-3 and significantly decreases specifically in responders	[29]
Panagopoulou (2019)	*Oncogene*	150 adjuvant, 16 neoadjuvant, 34 metastatic and 35 healthy volunteers	Methylation using a 5-gene panel (*KLK10*, *SOX17*, *WNT5A*, *MSH2*, *GATA3*) combined with cfDNA levels produced highly potent signatures to predict treatment response (AUC 0.803) and survival in metastatic settings.	[30]
Moss (2020)	*Ann. Oncol.*	33 patients with localized breast cancer and 64 healthy controls	Methylation signature (*KRT19*, *LMX1B*, *ZNF296*) allowed for universal detection with 80% sensitivity and served as a powerful indicator of residual disease towards the end of chemotherapy.	[31]
Kjær (2023)	*Sci. Rep.*	80 women with newly diagnosed, histologically verified breast cancer	Breast-specific (*LMX1B*, *ZNF296*) and cancer-specific (*HOXA9*) methylations showed increased sensitivity when combined but did not show a significant association with final pathological response.	[32]
Tokura (2024)	*Breast Cancer Basic Clin. Res.*	60 patients with HER2-positive early breast cancer (Target enrollment)	Study protocol established to evaluate the relationship between minimal residual disease (MRD) detection—using genetic profiles and DNA methylation—and clinical recurrence risk to guide treatment escalation or de-escalation.	[35]
Ravera (2024)	*J. Liq. Biopsy*	7 women with TNBC	Methylome patterns clearly differentiate patients achieving a pathological complete response (pCR) from those with residual disease.	[33]
Shan (2024)	*J. Liq. Biopsy*	44 TNBC	cfDNA levels and methylation changes effectively predict both the extent of residual cancer burden (RCB) and the risk of recurrence in TNBC patients.	[34]
Elliott (2025)	*ESMO Open*	95 early-stage ER+ or TNBC	Methylation-based MRD detection post-surgery is a potent prognostic factor for event-free survival (HR 17.0) and outperforms mutation tracking by providing a molecular lead time of several months before clinical relapse.	[36]
Panagopoulou (2026)	*Breast Cancer Res.*	195 LABC, 135 healthy individuals	Methylation (*CLDN15*, *MRGPRD*, *ZNF430*) can accurately predict treatment response (AUC 0.86) and relapse (AUC 0.79).	[37]

**Table 4:** Overview of literature aimed at evaluation of ctDNA status.

Paper	Journal	Patient Cohort	Major Finding(s)	Ref.
Radovich (2020)	*JAMA Oncol.*	196 TNBC who had residual disease after neoadjuvant chemotherapy	Detection of ctDNA after neoadjuvant chemotherapy and surgery was independently associated with significantly inferior distant disease-free survival (DDFS), disease-free survival (DFS), and overall survival (OS)	[40]
Valencia (2021)	*World J. Clin. Oncol.*	Case report of a 54-year-old female patient with stage IIIA triple-negative breast cancer (TNBC)	*PIK3CA* mutation in ctDNA after neoadjuvant treatment and surgery might be associated with early disease relapse or rapid disease progression	[41]
Wei (2022)	*Appl. Biochem. Biotechnol.*	56 LABC	Baseline ctDNA mutation of *XRCC1* was significantly associated with good neoadjuvant chemotherapy efficacy, while *mTOR* mutation was potentially associated with resistance	[42]
Stecklein (2023)	*npj Breast Cancer*	80 TNBC with residual disease after neoadjuvant systemic therapy	End-of-treatment ctDNA status is independently prognostic in TNBC patients with residual disease; ctDNA positivity was significantly associated with inferior 3-year event-free survival (EFS) and overall survival (OS)	[38]
Kim (2023)	*JNCI J. Natl. Cancer Inst.*	207 metastatic, 465 stage II-III TNBCs	High baseline ctDNA copy number aberration (CNA) burden, predicts poor disease-free survival (DFS) independently of pathologic complete response (pCR) in TNBC patients	[43]
Zaikova (2024)	*npj Breast Cancer*	130 TNBC (64 neoadjuvant and 66 adjuvant treatment cases)	Evaluation of actionable ctDNA mutations within seven months of completing primary treatment can identify a subgroup of TNBC patients at high risk of reduced progression-free survival, especially when combined with incomplete pathologic response	[44]
Du (2024)	*BMC Women’s Health*	231 LABC	Significant decreases in ctDNA levels during neoadjuvant chemotherapy correlate with achieving pathological complete response (pCR) and improved 3-year disease-free survival	[45]

**Table 5:** Overview of literature describing longitudinal ctDNA monitoring.

Paper	Journal	Patient Cohort	Major Finding(s)	Ref.
Garcia-Murillas (2015)	*Sci. Transl. Med.*	55 early BC	ctDNA detection in plasma after curatively intended treatment accurately identifies minimal residual disease and predicts metastatic relapse.	[46]
Riva (2017)	*Clin. Chem.*	38 TNBC	ctDNA positivity after just one cycle of neoadjuvant chemotherapy is significantly correlated with shorter disease-free and overall survival.	[47]
Kim (2017)	*Oncotarget*	33 LABC	Targeted ultra-deep sequencing of ctDNA more precisely indicates tumor biology and response to treatment than traditional tumor biopsy.	[48]
Butler (2019)	*Cold Spring Harb. Mol. Case Stud.*	10 LABC	Marked increases in mid-treatment ctDNA levels can serve as early predictors of disease progression and future recurrence.	[49]
Rothe (2019)	*Clin. Cancer Res.*	69 HER2-amplified BC	ctDNA detection prior to neoadjuvant therapy is associated with significantly decreased odds of achieving a pathologic complete response (pCR).	[50]
Garcia-Murillas (2019)	*JAMA Oncol.*	101 early-stage BC	Monitoring ctDNA during follow-up is highly prognostic for future relapse across all major subtypes, offering a 10.7-month median lead time over clinical recurrence.	[51]
McDonald (2019)	*Sci. Transl. Med.*	33 stage I-III BC	ctDNA concentrations after neoadjuvant therapy are significantly higher in patients with residual disease at surgery compared to those achieving pCR.	[52]
Cavallone (2020)	*Sci. Rep.*	26 TNBC	ctDNA detection early in treatment and late before surgery strongly predicts residual tumor, while presence at the end of NAC indicates worse relapse-free survival.	[54]
Hao (2020)	*J. Breast Cancer*	31 BC	Identifying specific somatic mutations, such as *KMT2C*, in ctDNA samples six months after surgery may serve as an indicator of disease recurrence.	[55]
Li (2020)	*JCO Precis. Oncol.*	52 early BC	Longitudinal ctDNA tracking outperforms traditional imaging in predicting neoadjuvant response and provides strong prognostic value for survival.	[56]
Magbanua (2021)	*Ann. Oncol.*	84 high-risk early BC	Early clearance of ctDNA predicts favorable response, whereas persistence during neoadjuvant therapy identifies patients unlikely to achieve pCR.	[57]
Ortolan (2021)	*ESMO Open*	42 TNBC	ctDNA detection after neoadjuvant therapy is associated with a high risk of relapse, predating clinical diagnosis by up to 13 months.	[58]
Zhou (2021)	*Breast Cancer Res. Treat.*	32 LABC	Sequential ctDNA monitoring is effective for evaluating treatment efficacy; post-operative ctDNA status is the strongest prognostic factor for metastasis.	[59]
Zhou (2022)	*Clin. Cancer Res.*	193 BC	Persistence of ctDNA midway through neoadjuvant therapy negatively predicts response and identifies patients at high risk for significant residual cancer burden.	[60]
Cailleux (2022)	*JCO Precis. Oncol.*	44 BC	ctDNA detection after neoadjuvant therapy and before surgery is strongly associated with shorter event-free survival.	[61]
Turner (2023)	*Ann. Oncol.*	161 TNBC	Prospective surveillance can identify molecular residual disease not visible on imaging, although high rates of metastatic disease are often found upon first detection.	[62]
Magbanua (2023)	*Cancer Cell*	283 HER2-negative BC	Early ctDNA clearance predicts response in TNBC, while ctDNA positivity at any time point is associated with inferior survival outcomes across subtypes.	[63]
Parsons (2023)	*Ann. Oncol.*	68 TNBC	Ultrasensitive enrichment assays show a strong association between end-of-therapy ctDNA levels and residual cancer burden status.	[64]
Liu (2023)	*BMC Med.*	269 BC	Incorporating pretreatment ctDNA levels significantly improves pCR prediction models; positive ctDNA after therapy predicts worse survival.	[65]
Garcia-Murillas (2025)	*Breast Cancer Res. Treat.*	61 early BC	ctDNA detection during monitoring is associated with a 100% positive predictive value for future disease relapse.	[66]
Elliott (2025)	*Nat. Commun.*	119 early BC	Mid-treatment ctDNA detection enhances residual cancer burden prognostication, and postoperative detection independently predicts recurrence.	[67]
Ademuyiwa (2025)	*Clin. Cancer Res.*	119 TNBC	A tissue-free epigenomic assay demonstrates high sensitivity and specificity for predicting distant recurrence during and after therapy.	[68]
Dong (2025)	*Breast Cancer Res.*	73 stage II/III BC	Undetectable ctDNA in longitudinal samples is associated with prolonged survival and accurately reflects neoadjuvant treatment efficacy.	[69]
Lin (2026)	*Cancer Res. Commun.*	117 HER2-positive BC	ctDNA persistence after neoadjuvant therapy independently predicts recurrence and may help determine the need for adjuvant T-DM1 therapy.	[70]
Park (2026)	*Breast Cancer*	119 BC	ctDNA non-clearance is the strongest independent prognostic factor for disease progression, with the most pronounced effect in TNBC.	[71]
Grinshpun (2026)	*ESMO Open*	52 HR-positive/HER2-negative BC	Pre-treatment ctDNA detection is associated with higher pathological stages, while persistent detection predicts a higher risk of recurrence.	[72]

## Data Availability

No new data were created or analyzed in this study. Data sharing is not applicable to this article.

## Abbreviations

The following abbreviations are used in this manuscript:

cfDNA	cell-free DNA
ctDNA	Circulating tumor DNA
NACT	neoadjuvant chemotherapy
UIM	Unique molecular identifier
ARMS	Amplification-refractory mutation system
ddPCR	digital droplet PCR
